# Static and Dynamic Correlations in Binary and Ternary Mixtures of TMAO, Urea, and Water

**DOI:** 10.1021/acs.jpcb.5c03841

**Published:** 2025-07-24

**Authors:** Christoph Hölzl, Dominik Horinek

**Affiliations:** Institut für Physikalische und Theoretische Chemie, 9147 Universität Regensburg, 93040 Regensburg, Germany

## Abstract

Force field molecular dynamics simulations of aqueous solutions of TMAO and urea are used to investigate the delicate interactions in binary and ternary mixtures of one of the most important osmolyte systems. We explore the effect of the choice of force fields on local interactions and thermodynamics. Fully decomposed dielectric relaxation spectra from simulations are used to interpret existing experimental data and evaluate currently used fitting techniques. We show that many force field combinations describe the potential of mean force between urea and TMAO, but it is more challenging to describe thermodynamic data for the ternary system like activity coefficients.

## Graphical abstract

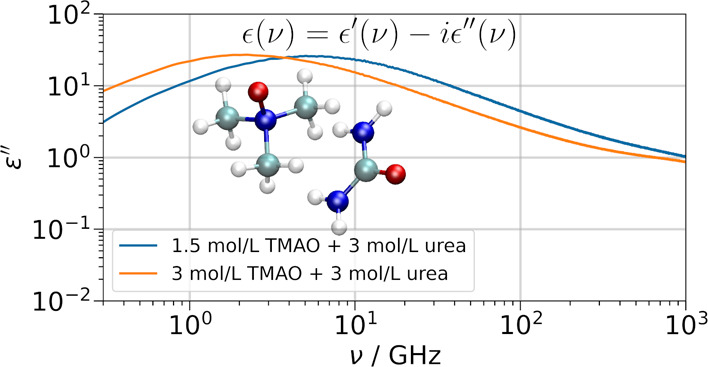

## Introduction

Osmolytes are small molecules that perform biologically relevant functions in the cytoplasm of many organisms. In addition to their osmoregulatory function, osmolytes can also affect the stability of proteins against external strains.^1^ The class of osmoprotectants, such as methylamines, glycine, and glycine betaine, stabilize proteins, among other factors, against temperature or pressure denaturation, whereas denaturants such as urea, arginine, and guanidinium lead to preferential unfolding of proteins.^2,3^ It has been found in vitro that for the protectant trimethylamine–N–oxide (TMAO)^4^ and the denaturant urea, which are present in deep-sea fish at a molar ratio of 2:1, TMAO counteracts the denaturing effect of urea in an approximately additive manner.^5,6^ This is the most widely studied pair of osmolytes, and their individual effects on proteins have been intensely investigated.

TMAO forms strong hydrogen bonds with approximately three water molecules,^7–9^ thus leading to a water structure that is less prone to pressure–induced perturbations.^10^ It is currently believed that, instead of a mainly indirect mechanism through water binding,^11,12^ TMAO stabilizes proteins through favorable exclusion from the backbone,^13,14^ which has been described as a “frustration mechanism” caused by a combination of the molecule’s large dipole moment and bulky hydrophobic groups.^15^ However, due to the accumulation of TMAO in the first hydration shell around peptides, an indirect influence on peptide–water hydrogen bonds can not be ruled out.^16^

Urea, on the other hand, predominantly interacts directly with the peptide backbone through favorable van der Waals interactions, which biases proteins toward unfolded states with a greater accessible surface area for urea–backbone interactions.^17–19^

The interplay of the interactions of TMAO and urea with each other, with water, and with proteins is still far from being fully understood. It has been found in X–ray scattering experiments that the hydration structure of both osmolytes changes very little between their binary and the ternary solution up to high concentrations.^20^ Neutron scattering experiments lead to the conclusion that, while TMAO and urea do interact directly, their interaction is too weak to be relevant at physiological concentrations.^21,22^ It was also concluded that TMAO causes a depletion of urea in the solvation shell of proteins.^23^

Early simulations propose that TMAO prevents urea–protein interactions by strongly binding urea through H–bonds,^24^ which agrees with results from Raman spectroscopy at high concentrations.^25^ However, a combined ab initio molecular dynamics and pump–probe IR spectroscopy study contradicts this claim and proposes that TMAO and urea interact favorably through hydrophobic interactions, and that TMAO favors water over urea as an H–bonding partner.^26^ The same qualitative behavior was found for different combinations of force field models.^27^ The picture of the binding of urea by TMAO is also corroborated by simulations of polyalanine and the R2 fragment of the Tau protein in binary and ternary osmolyte solutions, which find that the addition of TMAO reduces the preferential solvation of these peptides by urea.^28^

Many arguments on the precise nature of their interactions is based on classifying osmolytes as either structure formers (kosmotropes) or breakers (chaotropes), where structure specifically refers to the H–bond network of water.^11^ Recent simulations have begun to question this partition of osmolytes into kosmotropes and chaotropes by demonstrating that both TMAO and urea strengthen the H–bond network in binary and ternary solutions without forming a significant amount of solute–solute H–bonds.^29^

Overall, there exist many contradictory or ambiguous interpretations of the mechanistic behavior of TMAO and urea. A major question remains whether some of the contradictions between simulations are specifically due to deficiencies in the available force field representations. In this work, we aim to answer this question by investigating the influence of the choice of the urea model, which has been neglected for a long time since the introduction of the KBFF force field.^30^ We compare the Kirkwood–Buff integrals^31,32^ and the resulting activity data of simulations using a new urea model^33^ to experimental data and previous simulations.^27,28^

This model is further characterized by calculating the dynamics of binary and ternary systems in the form of dielectric relaxation spectroscopy (DRS), which is an important technique for directly analyzing the dynamics of liquids. Among other systems, it has been extensively applied to water and aqueous solutions.^34^ DRS measures the first-order rotational relaxation of a system’s total dipole moment. In this work, we calculate the dielectric relaxation spectra of concentrated binary and ternary aqueous solutions of TMAO and urea and compare them to broadband experimental spectra. We demonstrate that the molecular–level decomposition of the simulated spectra, which is inaccessible in DRS experiments, provides essential interpretations of the experimental spectra and can assist in their complex fitting procedure.

By characterizing a specific combination of models for TMAO, urea, and water, we show that it is possible to reproduce both static and dynamic properties, even for complex ternary systems.

## Methods

### Force Fields

Nonpolarizable models for TMAO have evolved over the years. One line of force field development was the Kast model,^35^ which was followed by the Netz model.^15^ Based on the Netz model, a pressure–dependent model was developed,^36^ hereafter called the HMKH model. It accurately reproduces experimental densities and activity coefficients of binary TMAO-water solutions at ambient conditions over a large concentration range, as well as radial distribution functions and H–bond distributions from ab initio simulations.^36^ Further Kast–based models were developed to reproduce experimental osmometry data (Garcia model)^37^ and the density of aqueous solutions (Shea model).^38^ Recently, the Netz model was modified for ternary TMAO–urea solutions by explicit scaling of the TMAO–TMAO and TMAO–urea interactions, called the Netz­(m) model.^28^

For urea, the most widely used model is the Kirkwood–Buff force field (KBFF),^30^ which accurately reproduces the activity data in aqueous solutions. We have recently shown that the solvation and hydrogen bonding structure of the KBFF model does not agree with predictions by ab initio molecular dynamics.^33^ In the same work, we developed a model through global optimization with the objective of reproducing the coordination and H–bond numbers from ab initio simulations and experimental densities over a large concentration range. The resulting model predicts activity data with the same accuracy as the KBFF model, and it significantly improves the performance with respect to many other properties. This model will hereafter be called the HMKH model.

In this work, we use the Netz­(m) and HMKH models for TMAO, the KBFF and HMKH models for urea, and the SPC/E^39^ and TIP4P/2005 ^40^ models for water. The Netz­(m) and KBFF models were developed for SPC/E, whereas the HMKH TMAO and urea models were originally optimized for TIP4P/2005. However, the HMKH TMAO model performs very well with the SPC/E water model^41^ and the properties of the KBFF urea model are similar in SPC/E and TIP4P/2005.^42^

### Simulation Details

All simulations were performed using the GROMACS 2021.5 package.^43^ The equations of motion were integrated by using the leapfrog integrator with a time step of 2 fs. All bond lengths were constrained using SETTLE^44^ for water and LINCS^45^ for TMAO and urea. For the van der Waals interactions, a Lennard–Jones potential with a cutoff of 1 nm was used together with the standard analytical tail correction to the energy and virial. The parameters for different atom types were calculated using Lorentz–Berthelot rules ( $\sigma_{ij}=\left(\sigma_{i}+\sigma_{j}\right)/2,\epsilon_{ij}=\sqrt{\epsilon_{i}\epsilon_{j}}$ ) for all systems except for those with the KBFF urea model, which is optimized for geometric combination rules ( $\sigma_{ij}=\sqrt{\sigma_{i}\sigma_{j}},\epsilon_{ij}=\sqrt{\epsilon_{i}\epsilon_{j}}$ ). We note that it has been shown that the effect of combination rules on ternary TMAO/urea/water systems is negligible.^27^ Electrostatic interactions were modeled with a Coulomb potential with a 1 nm real–space cutoff, and long-range interactions were calculated using smooth particle–mesh Ewald summation.^46^ For temperature control, we used the stochastic velocity rescaling thermostat^47^ with a coupling constant of 1 ps and a temperature of 300 K. Pressure was controlled using the Parrinello–Rahman barostat^48^ with a time constant of 2 ps. For pressure equilibration, the Berendsen barostat^49^ was used with a time constant of 1 ps.

For the calculation of Kirkwood–Buff integrals and activity coefficients, ternary systems with cubic box lengths of 4 nm were each simulated for 500 ns in the NpT ensemble after successive equilibration with Berendsen and Parrinello–Rahman barostats for 0.5 and 5 ns, respectively. The positions were written every 500 fs. The TMAO/urea concentrations in mol/L are 1/1, 1/2, 2/4, and 2.5/2.5.

Potentials of mean force between TMAO and urea were calculated via umbrella sampling for a system containing 1 TMAO, 1 urea, and 1073 water molecules with a box length of approximately 3.2 nm. A harmonic restraint potential with a force constant of 1000 kJ/mol/nm^2^ was applied to the distance between the TMAO oxygen and the urea carbon atoms, in order to be consistent with the methodology in ref 26. 50 windows with potentials centered between 0.24 and 1.24 nm were simulated in the NpT ensemble for 50 ns each. The restraint distance was written every 10 steps, and the potentials of mean force were calculated using the weighted histogram analysis method as implemented in GROMACS.^50^

The systems for the calculation of dielectric relaxation spectra were smaller, with cubic box lengths of 2.5 nm. We found no significant finite size effect on the spectra when compared to a 4 nm box (see Figure S1 in the Supporting Information). First, the density of the system was converged by equilibration with a Berendsen barostat for 500 ps. Then, the average box size was calculated from a 5 ns run by using the Parrinello–Rahman barostat. At the converged volume, the systems were first equilibrated in the NVT ensemble for 20 ns, and then run for up to 1.6 μs to generate independent starting conformations, including velocities, every 100 ps. Depending on the system, between 1000 and 16,000 trajectories of 4 ns (4–64 μs) were run in the NVT ensemble with an output frequency of 4 fs.

All system compositions, concentrations, and total sampling times are listed in Table S5 in the Supporting Information.

### Kirkwood–Buff Integrals and Activity Coefficients

The Kirkwood–Buff integrals^31^ (KBIs) *G* between species *i* and *j* are defined as

$$G_{ij}=\int_{0}^{\infty }dr4\pi r^{2}[g_{ij}^{\mu VT}(r)-1]$$ 1

Here, *g*_*ij*_^*μVT*^(*r*) is the radial distribution function (RDF) between molecular centers of mass in the grand canonical ensemble, for which this expression is exact. We corrected the radial distribution functions from simulations of closed systems in the NpT ensemble using the empirical expression of Ganguly and van der Vegt:^51^

$$g_{ij}^{\mathrm{corr}}(r)=g_{ij}^{\mathrm{NpT}}(r)\frac{N_{j}\left(1-\frac{4\pi r^{3}}{3V}\right)}{N_{j}\left(1-\frac{4\pi r^{3}}{3V}\right)-\delta_{ij}-\frac{N_{j}}{V}\int_{0}^{r}dr4\pi r^{2}[g_{ij}(r)-1]}$$ 2

Here, *N*_*j*_ is the total particle number of species *j*, *V* is the system volume, and δ_*ij*_ is the Kronecker delta. The KBIs in a ternary system are connected to the concentration derivatives of the chemical potentials^32^

$${\left(\frac{\partial \mu_{i}}{\partial c_{i}}\right)}_{T,P}=\frac{kT\left(c_{j}+c_{k}+c_{j}c_{k}\Delta_{jk}\right)}{\eta c_{i}}$$ 3

with the chemical potentials μ, the molar concentrations *c*, and the auxiliary quantities Δ_*ij*_ and η:

$$\Delta_{ij}=G_{ii}+G_{jj}-2G_{ij}$$ 4

$$\begin{array}{rll} \eta & =c_{i}+c_{j}+c_{k}+c_{i}c_{j}\Delta_{ij}+c_{j}c_{k}\Delta_{jk}+c_{i}c_{k}\Delta_{ik} & \\ & -\frac{1}{4}c_{i}c_{j}c_{k}\left(\Delta_{ij}^{2}+\Delta_{jk}^{2}+\Delta_{ik}^{2}-2\Delta_{ik}\Delta_{jk}-2\Delta_{ij}\Delta_{ik}-2\Delta_{ij}\Delta_{jk}\right) \end{array}$$ 5

Using the thermodynamic relation for the chemical potential with the standard chemical potential μ_*i*_^0^ and the molarity-scale activity coefficient *y*_*i*_

$$\mu_{i}=\mu_{i}^{0}+kT\ln\left(y_{i}c_{i}\right)$$ 6

an expression for the unitless logarithmic concentration derivative of the activity coefficient is obtained:

$${\left(\frac{\partial \mu_{i}}{\partial c_{i}}\right)}_{T,P}=kT\left({\left(\frac{\partial \ln y_{i}}{\partial c_{i}}\right)}_{T,P}+c_{i}^{-1}\right)$$ 7

$$y_{ii}={\left(\frac{\partial \ln y_{i}}{\partial \ln c_{i}}\right)}_{T,P}=\frac{c_{j}+c_{k}+c_{j}c_{k}\Delta_{jk}}{\eta }-1$$ 8

### Dielectric Relaxation Spectroscopy

The complex frequency–dependent electric permittivity is

$$\epsilon \left(\nu \right)=\epsilon^{\prime }\left(\nu \right)-i\epsilon^{\prime \prime }\left(\nu \right)$$ 9

Its negative imaginary part is the dielectric loss, which can be calculated within linear response theory^52^ as the Fourier–Laplace transform of the dipole current time autocorrelation function

$$\epsilon^{\prime \prime }(\nu )=\frac{1}{6\pi \epsilon_{0}k_{B}TV\nu }\int_{0}^{\infty }dt\exp(-i2\pi \nu t)\langle \dot{M}(0)\dot{M}(t)\rangle$$ 10

where ϵ_0_ is the vacuum permittivity, *k*_B_ the Boltzmann constant, *V* the volume, and **Ṁ** the time derivative of the total dipole moment of the system. Since **Ṁ** is the sum of the molecular contributions **μ̇**, the correlation function can be decomposed. For a single–component system, the decomposition is

$$\begin{array}{rll} \left\langle \dot{M}(0)\dot{M}(t)\right\rangle & =\left\langle \sum_{i=1}^{N}\sum_{j=1}^{N}{\dot{\mu }}_{i}(0){\dot{\mu }}_{j}(t)\right\rangle & \\ & =\sum_{i=1}^{N}\langle {\dot{\mu }}_{i}(0){\dot{\mu }}_{i}(t)\rangle +\sum_{i=1}^{N}\langle {\dot{\mu }}_{i}(0)[\dot{M}(t)-{\dot{\mu }}_{i}(t)]\rangle & \\ & =C_{\mathrm{self}}+C_{\mathrm{cross}} & \end{array}$$ 11

The first term is the autocorrelation of molecular dipoles, hereafter called the self-term, and the second term contains the molecular cross-correlations, hereafter called the cross-term. For two and three components α, β, and γ, the decompositions are

$$\langle \dot{M}(0)\dot{M}(t)\rangle_{\alpha \beta }=C_{\mathrm{self}}^{\alpha \alpha }+C_{\mathrm{cross}}^{\alpha \alpha }+C_{\mathrm{self}}^{\beta \beta }+C_{\mathrm{cross}}^{\beta \beta }+2C_{\mathrm{cross}}^{\alpha \beta }$$ 12

$$\left\langle \dot{M}(0)\dot{M}(t)\rangle_{\alpha \beta \gamma }=C_{\mathrm{self}}^{\alpha \alpha }+C_{\mathrm{cross}}^{\alpha \alpha }+C_{\mathrm{self}}^{\beta \beta }+C_{\mathrm{cross}}^{\beta \beta }+C_{\mathrm{self}}^{\gamma \gamma }+C_{\mathrm{cross}}^{\gamma \gamma }+2(C_{\mathrm{cross}}^{\alpha \beta }+C_{\mathrm{cross}}^{\alpha \gamma }+C_{\mathrm{cross}}^{\beta \gamma }\right)$$ 13

with the inter–species cross-correlations:

$$C_{\mathrm{cross}}^{\alpha \beta }=\left\langle \left(\overset{N_{\alpha }}{\underset{i=1}{\sum }}{\dot{\mu }}_{i}\left(0\right)\right)\left(\overset{N_{\beta }}{\underset{j=1}{\sum }}{\dot{\mu }}_{j}\left(t\right)\right)\right\rangle$$ 14

Due to linearity of the Fourier transform, the dielectric loss ϵ^*″*^ is decomposed in the same way.

For each 4 ns trajectory, the molecular dipole currents were calculated by using forward differences and then correlated by using FFT convolutions. The resulting correlation functions were then averaged over all trajectories, modified by a Hann window function^53^ over the full length, and finally Fourier–Laplace transformed to obtain the dielectric loss spectra.

## Results and Discussion

### Static Correlations: Kirkwood–Buff Integrals and Activity Coefficients

In Figure 1, we show the Kirkwood–Buff integrals (KBIs) for three force field combinations vs experimental data from inverted Kirkwood–Buff theory. We used the experimental data as well as the choice of concentrations from ref 27. Unsurprisingly, the Netz­(m) model for TMAO yields the best KBIs for almost all terms and concentrations, since it was specifically parametrized to reproduce the KBIs for ternary mixtures with KBFF urea and SPC/E water. It was created because the HMKH TMAO model, which works very well in binary aqueous solutions both with SPC/E and TIP4P/2005 water,^41^ when it is combined with the KBFF model for urea, fails to reproduce the KBIs in ternary solutions. Combining the HMKH TMAO and urea models yields KBIs with accuracy comparable to results from the system with the Netz­(m) force field. In some cases, like the UT term at 2 mol/L TMAO + 4 mol/L urea (Figure 1b), the HMKH//HMKH//TIP4P/2005 combination even outperforms Netz­(m)//KBFF//SPC/E. By design, the Netz­(m) model leads to the most accurate molar activity coefficient derivatives as calculated from eq 8 (Figure 2), but the values for both solutes, *y*_TT_ and *y*_UU_, deviate by approximately 0.15 from the experimental reference, which would lead to large errors in the integrated activity coefficients. This shows that there is a need for improvement, which the HMKH urea model can not solve due to deficiencies in the activity of binary urea–water mixtures at high concentrations.^33^ However, incorporating the solvation structure in addition to thermodynamic data in the parametrization avoids artifacts due to error compensation of van der Waals and electrostatic interactions, which is the case for the KBFF model.^33^

**Figure 1:**
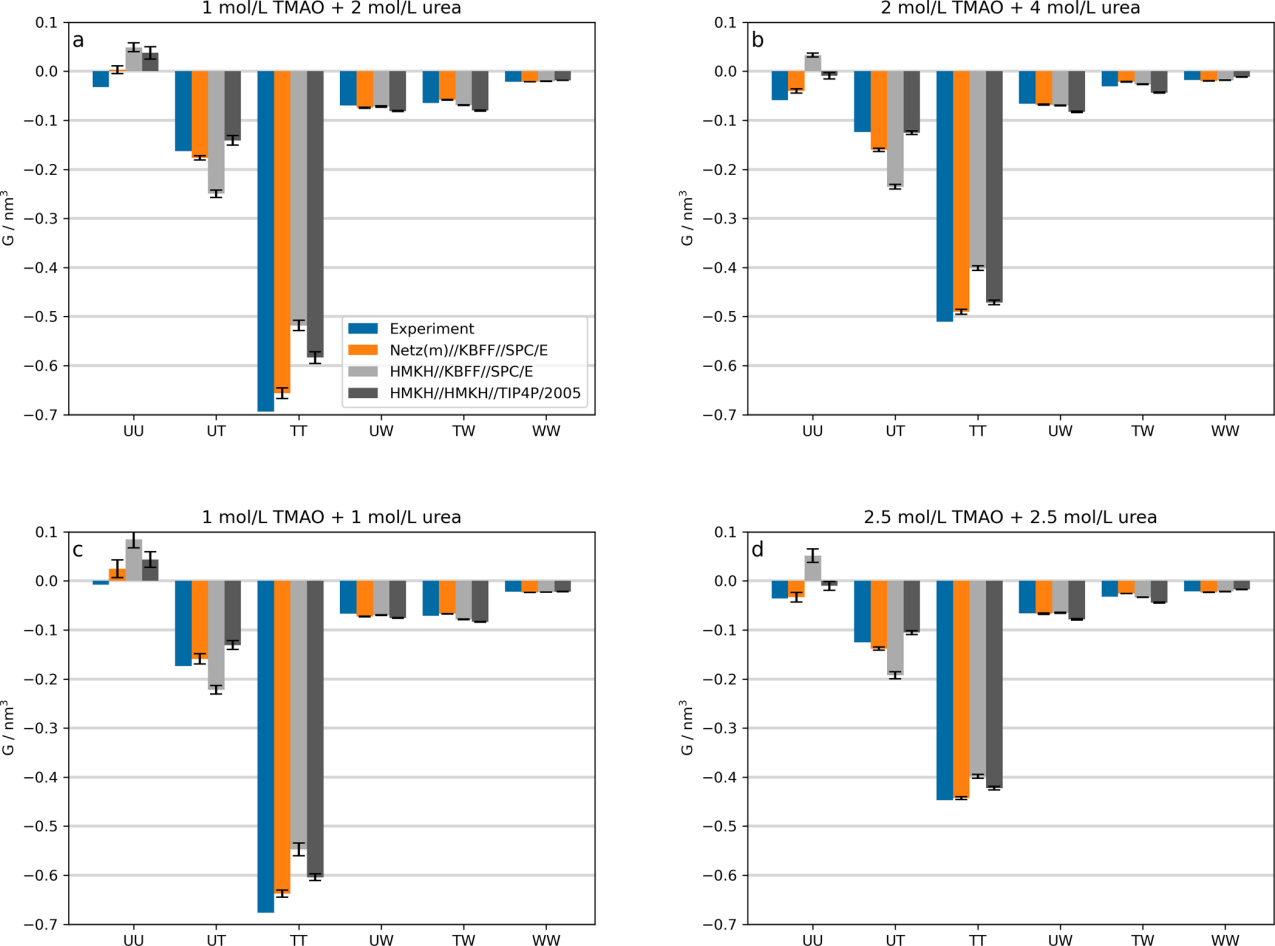
Kirkwood–Buff integrals for ternary TMAO/urea/water mixtures at different concentrations. Experimental data are taken from ref 27. KBIs from simulations were calculated as the averages in the range of 1.0–1.4 nm. Error estimates are calculated as the standard deviation of the mean of 10 trajectory blocks of 50 ns each.

**Figure 2:**
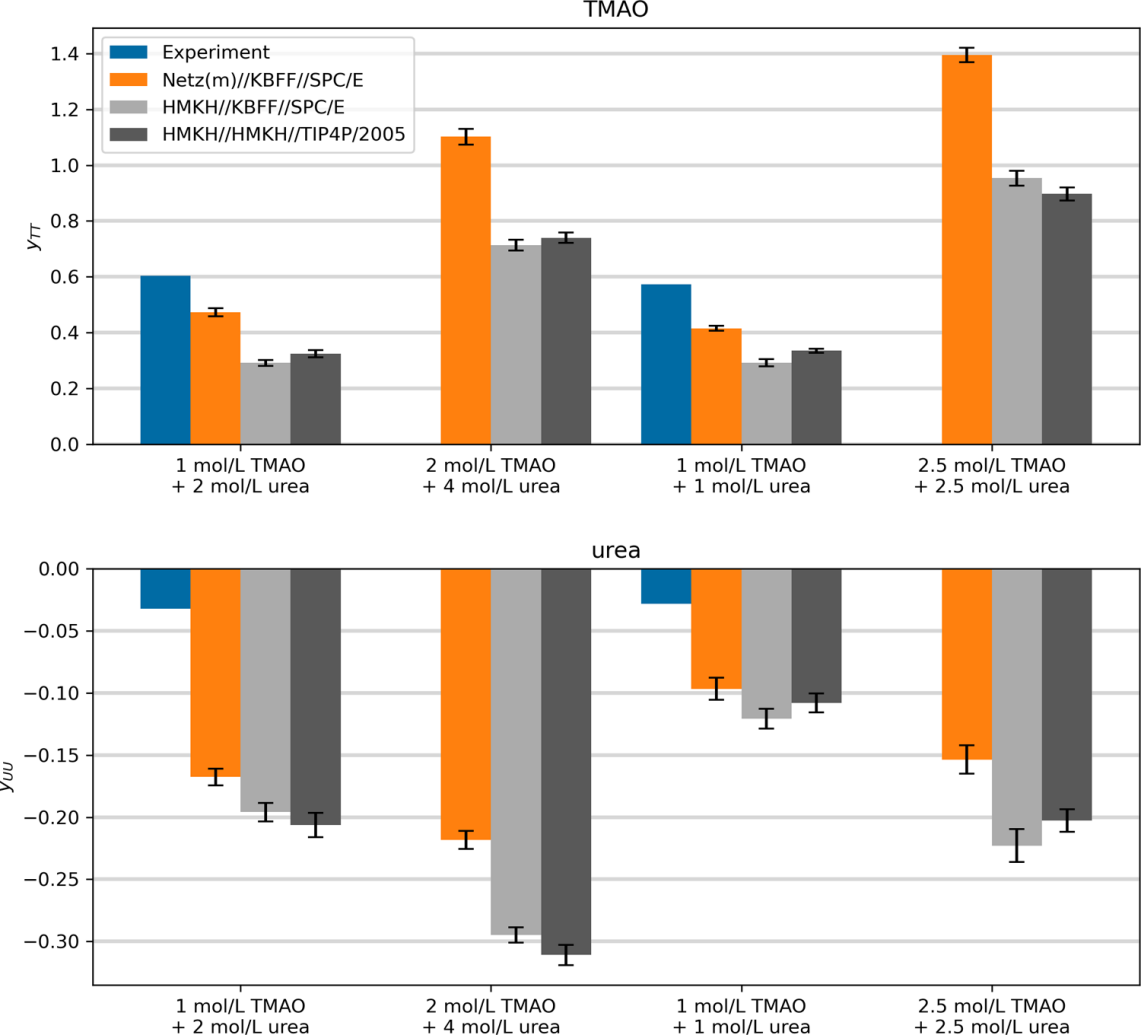
Logarithmic activity coefficient derivatives $y_{ii}=\frac{\partial \ln\;y_{i}}{\partial \ln\;c_{i}}$ for TMAO and urea in ternary solutions. Experimental data were calculated from the activity fit functions from ref 54 using the revised parameters from ref 27 and numerically differentiated to obtain concentration derivatives. The force field results were calculated by using eq 8.

Since a lot of work has already been done on very accurate TMAO models,^41^ further improvement will likely come from an improvement of force field representations for the urea molecule.

### Potential of Mean Force

The potential of mean force (PMF) between TMAO and urea as a function of the TMAO-O to urea-C distance has previously been calculated using ab initio molecular dynamics with the BLYP-D3 and revPBE-D3 functionals as well as the force field combination Kast (TMAO), OPLS (urea),^55^ and SPC/E (water).^26^ Since the OPLS model for urea aggregates in aqueous solutions instead of forming the expected, almost ideal solution,^56^ we will only discuss the KBFF^30^ and HMKH^33^ models for urea in this work. The ab initio data is shown together with several force field combinations in Figure 3. All models have a shallow global minimum at 0.56–0.57 nm in common, which corresponds to hydrophobic interactions between the TMAO methyl groups and urea. Only the HMKH//HMKH//TIP4P/2005 model reproduces the local minimum of the revPBE functional at 0.4 nm, which corresponds to hydrogen bonds between TMAO oxygen and urea hydrogens. We note that in the Supporting Information by Xie et al.,^26^ the confidence intervals of the PMFs with BLYP-D3 and revPBE-D3 overlap over the entire range of distances, which means that the existence of a local minimum due to H–bonds is not confirmed in AIMD. None of the force field combinations in this work result in a negative PMF in this region, which shows that all force fields qualitatively reproduce the behavior of AIMD, namely that the main favorable interactions are between the hydrophobic groups of TMAO and urea.^26^ Several other TMAO force fields, including the Netz model, display the same behavior as the KBFF and SPC/E models.^27^ Therefore, for currently used models, TMAO–urea interactions are qualitatively in line with AIMD results, even for combinations of force fields that were not specifically parametrized for this application. Still, the question remains whether the stabilizing/destabilizing effects of TMAO and urea approximately cancel due to direct or indirect, i.e., water–mediated, interactions.

**Figure 3:**
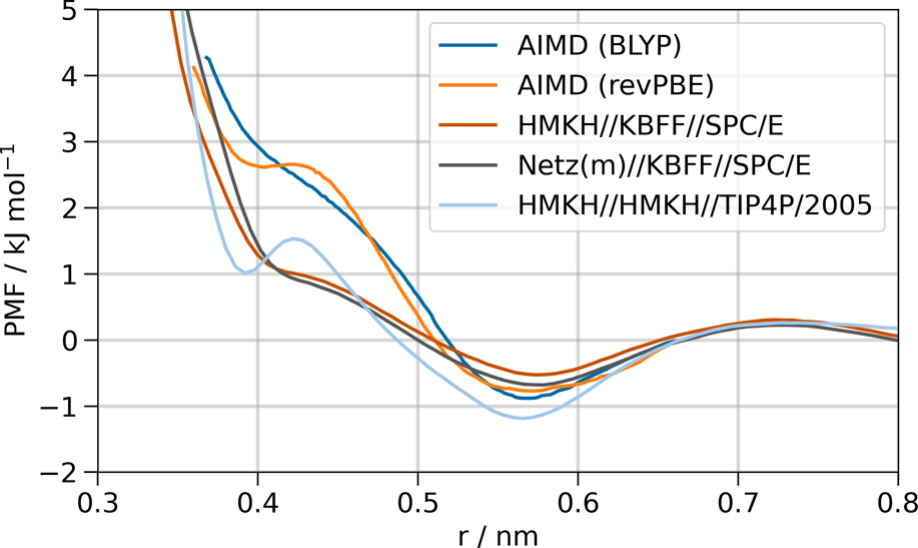
PMFs as a function of TMAO oxygen to urea carbon distance for a system containing 1 TMAO and 1 urea molecule. Ab initio molecular dynamics (AIMD) data was taken from ref 26. The force field simulation PMFs were calculated via umbrella sampling.

### Dynamic Correlations: Dielectric Relaxation Spectroscopy

We calculated the full decomposition of the dielectric loss spectra from simulations of binary and ternary solutions of TMAO and urea. Recently, the simulated spectra of the binary systems were decomposed by species,^57^ but we have, for the first time, performed the much more expensive decomposition into intra-species molecular dipole auto- and cross-correlation terms for these osmolyte systems. This molecular–level decomposition is critical for a correct interpretation of the spectra.

The simulated spectra were compared to experimental broadband dielectric spectra.^7,58^ In experiments, only the total dielectric loss is directly accessible, which is commonly fitted by using a sum of Debye functions, which have a Lorentzian function as the imaginary term. Physically, a Debye function corresponds to a fully uncorrelated rotational diffusion process of the molecular dipoles, i.e. a monoexponential dipole correlation function with relaxation time τ and maximum intensity at frequency ν^max^ = (2πτ)^−1^. However, water reorientation occurs through a jump mechanism,^59–61^ and the fit of the dielectric relaxation in bulk water by a single Debye function centered around 20 GHz^62,63^ does not correctly account for the fact that the main Debye–like loss peak is the sum of molecular auto– and cross-correlations.^64^ Nonetheless, Debye fits are the de facto standard even for more complex systems, such as binary and ternary TMAO/urea/water solutions. At least for aqueous urea, the rotational relaxation of urea molecules seems to be diffusive, thereby justifying the use of a Debye model for the solute.^65^ In the following chapter, we compare the fully decomposed spectra from simulations according to eqs 11–13 to experimental data^7,58^ at 3 mol/L (Figures 4–6). All spectra and their decompositions are shown in Figures 4–7.

**Figure 4:**
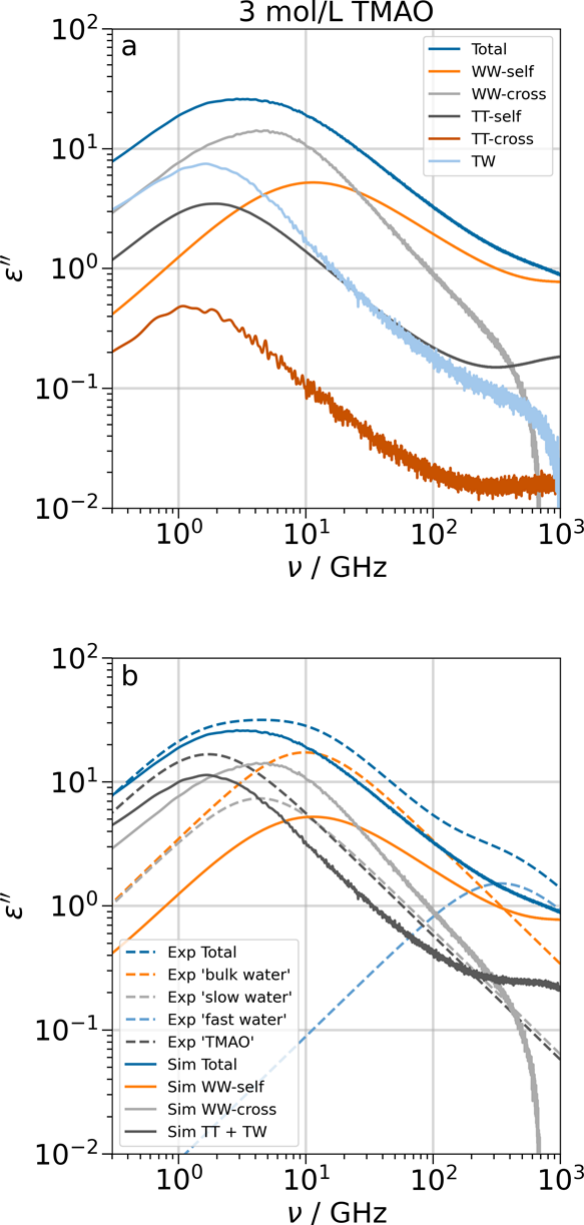
Simulated spectra (full lines) and Debye fits of experimental data for 3 mol/L TMAO^7^ (dashed lines). Panel a is the full decomposition of the simulated spectra. In panel b, the terms from simulations are combined to most closely reproduce the components in the fits of experimental data. TT in panel b is the sum of the TMAO–TMAO self- and cross terms.

**Figure 5:**
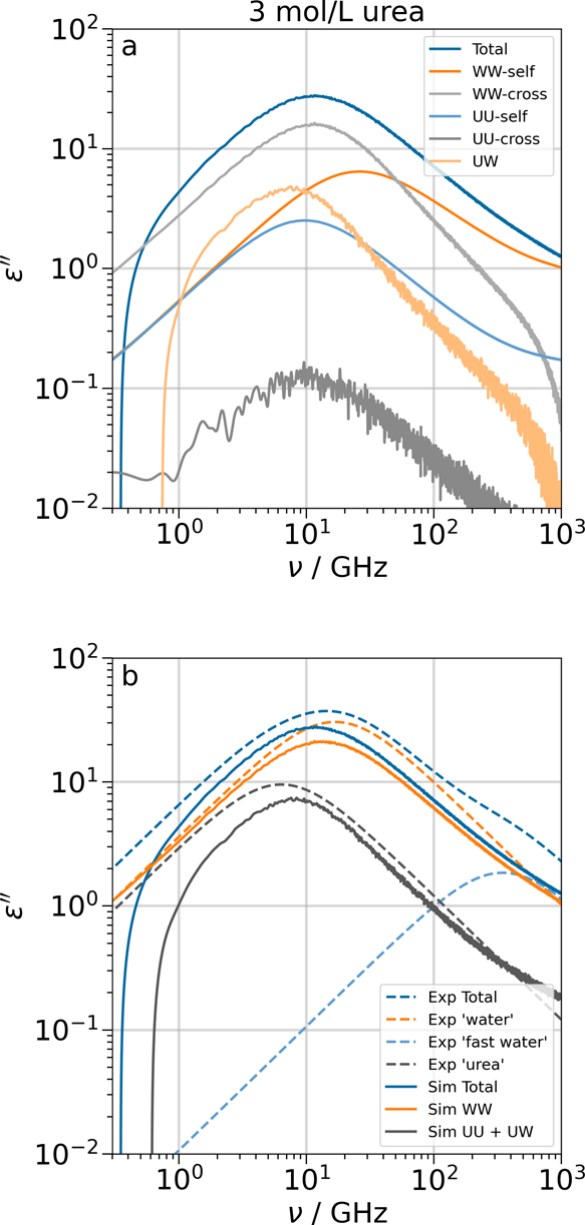
Simulated spectra (full lines) and Debye fits of experimental data for 3 mol/L urea^58^ (dashed lines). Panel a is the full decomposition of the simulated spectra. In panel b, the terms from simulations are combined to most closely reproduce the components in the fits of experimental data. UU in panel b is the sum of the urea–urea self-and cross terms.

**Figure 6:**
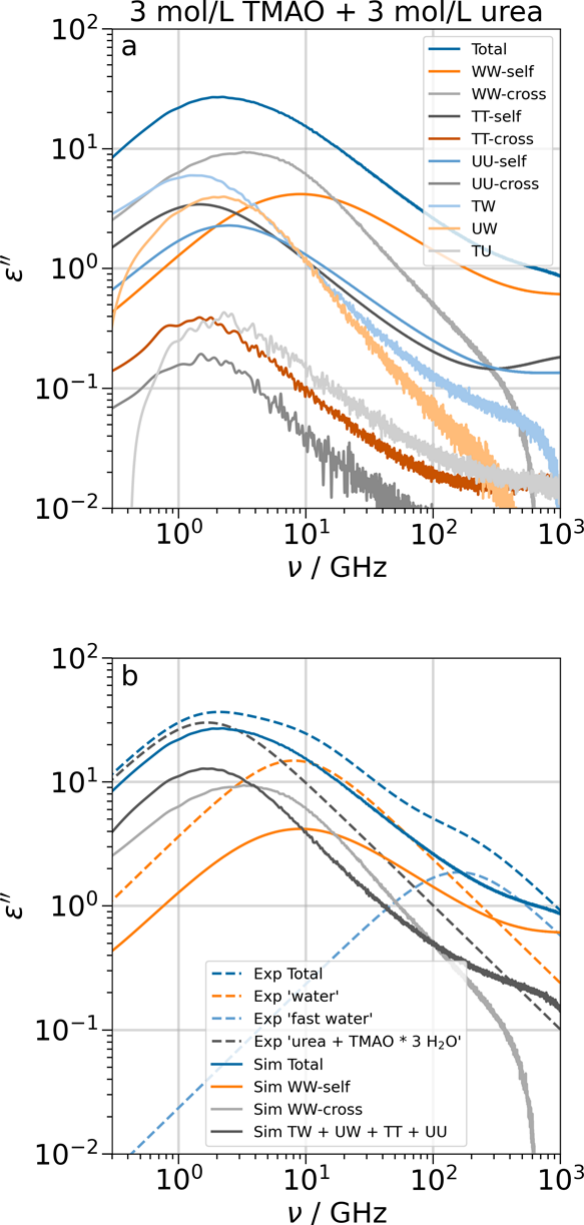
Simulated spectra (full lines) and Debye fits of experimental data for the ternary equimolar system at 3 mol/L^58^ (dashed lines). Panel a is the full decomposition of the simulated spectra. In panel b, the terms from simulations are combined to most closely reproduce the components in the fits of experimental data. TT and UU in panel b are the sum of TMAO–TMAO or urea–urea self-and cross terms, respectively.

**Figure 7:**
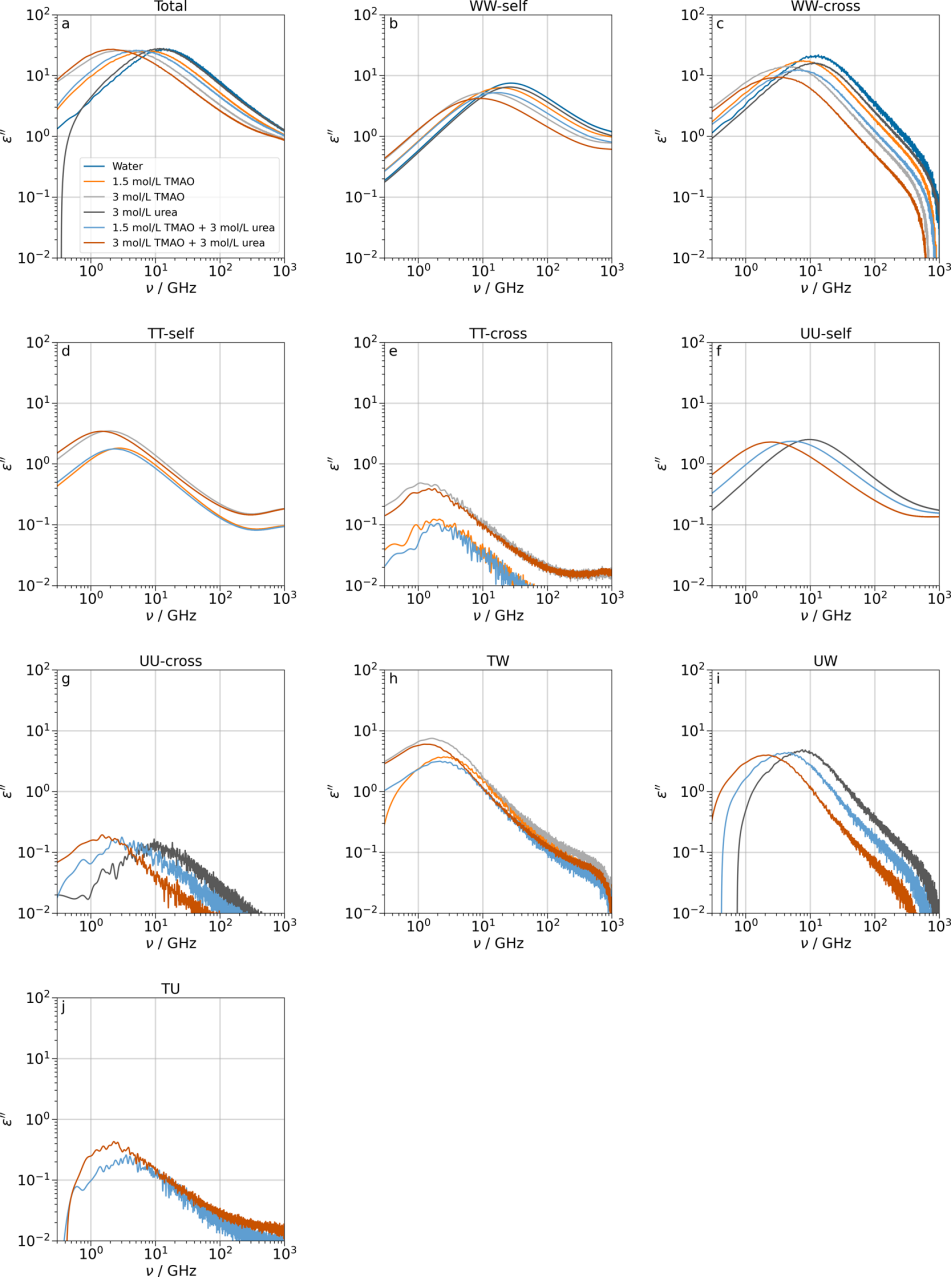
Simulated components of the dielectric loss for different concentrations of binary and ternary TMAO–urea–water systems. The molecular dipole contributions have been decomposed according to eqs 11 – [Disp-formula eq13].

#### Binary TMAO/Water Solution

In the spectral decomposition of a 3 mol/L TMAO solution in Figure 4a, we see that the dynamics of water is significantly slower compared to bulk, with the self- and cross terms centered at 11 and 5 GHz, which yields a total water peak at 6 GHz (compared to approximately 20 GHz in pure bulk water; see Figure 7a). The peak positions of the TMAO–TMAO self- and cross-terms, as well as the TMAO-water term, are very close and lie in the range 1.4–1.9 GHz. We also observe that even in highly concentrated TMAO solutions, the contribution from TMAO–TMAO cross-correlations is negligible. This is an indication that TMAO molecules have no propensity to interact with each other, which is in line with TMAO being an osmolyte. In order to simplify the decomposition into fewer terms, it is reasonable to combine all terms involving TMAO into one, which is shown in Figure 4b.

In ref 7, the dielectric spectra are fitted using a four-Debye model with the terms ‘TMAO’, ‘slow water’, ‘bulk water’, and ‘fast water’. The decomposition of simulations (Figure 4b) shows that the total loss agrees qualitatively, but the intensity is too low. This is largely due to the low dielectric loss of the TIP4P/2005 water model.^66^ The position of the ‘TMAO’ Debye component coincides with the sum of all TMAO–TMAO and TMAO-water terms. It is known that each TMAO forms on average three strong H–bonds to water molecules,^7,9,36^ which significantly slows down the rotation of the bound water molecules as well as the TMAO molecules. Both effects are represented by the TMAO–TMAO and TMAO-water correlations. The ‘slow water’ component is centered at the same frequency as the water–water cross-correlations, WW-cross. Since the dynamics of those water molecules that reside in the solvation shell around the bulky methyl groups of TMAO is also significantly slowed down due to the excluded volume caused by the hydrophobic cavities,^67^ it is reasonable to assume that the ‘slow water’ component describes this slower rotational relaxation process that is one contribution to the water–water cross-correlations. The ‘bulk water’ term appears to correspond to just the water–water autocorrelations WW-self, even though it appears at significantly lower frequencies than in bulk water.^62^ The fit of the experimental data includes a fourth Debye peak at 341 GHz that has a rather low intensity. These ‘fast water’ molecules have no correspondence in the decomposition of the spectra from simulations, and the necessity of a fourth peak might stem from the choice of Debye fits for non–Debye processes.

Quantitatively, the intensity of the ‘bulk water’ component is greater than the ‘slow water’ term. This is a different approach to the decomposition into auto- and cross-correlations in simulations, since the intensity of cross-correlations dominates the dielectric loss in water.

#### Binary Urea/Water Solution

In the case of 3 mol/L urea (Figure 5a), the peaks of the water self– and cross terms are at the same frequencies as in bulk water (see Figure 7b,c) at approximately 27 and 12 GHz respectively, which suggests that urea has almost no effect on water dynamics, which is in agreement with results from pump–probe IR spectroscopy.^68^ The urea–urea self- and cross terms are both centered at 10 GHz. As in the TMAO solution, the dielectric loss from solute–solute cross-correlations is negligible. The largest term involving urea is the urea-water relaxation centered at around 8 GHz. Again, it is a reasonable simplification to combine all urea terms into one component (Figure 5b). In ref 58, the experimental spectra of urea solutions are fit by a three-Debye model with the terms ‘urea’, ‘water’, and ‘fast water’. By splitting the simulated spectra into two terms, one containing all interactions involving urea and the other containing the water–water interactions, these terms accurately correspond to the experimental ‘urea’ and ‘water’ components, respectively. Thus, the experimental fit is a good description of the actual components that make up the dielectric loss, with the exception of the 'fast water' mode, as was discussed before.

#### Ternary TMAO/Urea/Water Solution

The water self- and cross terms in the ternary equimolar 3 mol/L solution (Figure 6a) are centered at 10 and 4 GHz, which is slower than in the binary TMAO solution and in the binary urea solution. The intensities of the solute–solute self-terms and solute-water correlations have the same order of magnitude and lie between 1.4 and 2.6 GHz. Again, the effect of solute–solute cross-correlations (1.5–2.8 GHz) on the dielectric loss is negligible. Thus, we include them together with all solute–solute and solute-water terms into one component. The experimental spectra of the ternary system were fitted by a model with three Debye functions that were designated as ‘urea + TMAO × 3 H_2_O’, ‘water’, and ‘fast water’. Our simulated spectrum (Figure 6b) differs in the total intensity but qualitatively reproduces the experimental data up to 100 GHz. We find that the component ‘urea + TMAO × 3 H_2_O’ corresponds to the sum of all TMAO–TMAO, urea–urea, TMAO-water, urea-water, and TMAO-urea interactions of the simulated spectra, even though the intensity of this combined peak is too low. The experimental ‘water’ peak is close to the simulated water–water self-term, but the intensities do not match. We propose that the discrepancies between experimental intensities of the ‘urea + TMAO × 3 H_2_O’ and ‘water’ peaks and their counterparts in simulations are due to contributions from the water–water cross-correlations, which are a separate contribution in our decomposition but contribute to both peaks of the experimental fits. Thus, a separate term for the water–water cross term, similar to the ‘slow water’ term in the binary TMAO system, might lead to a fit that is in agreement with our decomposition.

#### Concentration Effects

We have shown above that the decomposition of the water–water term into molecular dipole auto– and cross-correlations is essential for the understanding of the dynamics of multicomponent aqueous solutions, such as the osmolyte solutions in this work. Further insight comes from studies at different concentrations. We show the concentration-dependent effect of TMAO and urea on all components of the dielectric loss in Figure 7. The main observation is that any increase in solute concentration slows down the dynamics of all components. Although the correlations between urea and water are relatively strong (Figures 5, 6, and 7i), the influence of urea on the dynamics of water is small in binary mixtures and the ternary systems (Figure 7b,c). As mentioned before, the strong hydrogen bonds formed by TMAO to water and its bulky hydrophobic groups significantly disturb the dynamics of water (Figure 7h). Adding TMAO to urea solutions or vice versa also slows the dynamics of both solutes compared to their binary solutions (Figure 7d–g). The potential of mean force displays no short–ranged favorable H–bonding interactions between TMAO and urea, which is in agreement with ab initio simulations.^26^ Therefore, the retardation is most likely due to both direct hydrophobic TMAO–urea and indirect water–mediated interactions. Although the contribution of solute–solute cross-correlations to the total dielectric loss is negligible, they follow the same concentration effect trends as the solute-water and water–water dynamics.

## Conclusions

The full decompositions of simulated dielectric relaxation spectra show that Debye fits of experimental data can qualitatively describe the low-frequency contributions. However, more detailed information and more flexible fit functions are required for a better description of these complex dielectric relaxation processes. We found that many force field combinations accurately describe the PMF between urea and TMAO from ab initio methods. However, the analysis of Kirkwood-Buff integrals shows that it is still challenging to obtain accurate activity coefficients for ternary systems. The development of TMAO force fields has been addressed in several studies within the past decade, which led to force fields that perform satisfactorily with respect to structure and dynamics. For urea, the KBFF force field, which has its weaknesses in the reproduction of the detailed solvation structure, has been state-of-the-art. This property is better reproduced by our recently developed HMKH force field.^33^ Still, the urea force fields are the most likely candidates for significant improvements to the solvation structure and thermodynamic properties of ternary systems.

## Supplementary Material



## Notes

Force field parameters for TMAO and urea; system compositions; finite size effects; relation between dipole and current autocorrelation fucntions; KBI error estimates; (PDF)
