# Supplementary material for: Static and Dynamic Correlations in Binary and Ternary Mixtures of TMAO, Urea, and Water
Source: J Phys Chem B. 2025 Jul 24;129(31):7972–81. doi: 10.1021/acs.jpcb.5c03841 (PMC12337090; doi:10.1021/acs.jpcb.5c03841)
Supplement: Supplementary file 1 [file jp5c03841_si_001.pdf]

Supporting Information: Static and Dynamic Correlations in Binary and Ternary Mixtures of TMAO, Urea, and Water

Christoph Hölzl and Dominik Horinek
Institut für Physikalische und Theoretische Chemie, Universität Regensburg, 93040 Regensburg, Germany

S1 Force field parameters

The TMAO and urea force fields in this work use the following potentials for bonded interactions:

$$V_{\text{angle}}(\phi) = \frac{k_\phi}{2}(\phi - \phi_0)^2 \tag{S1}$$

$$V_{\text{dihedral}}(\theta) = k_\theta(1 + \cos(n\theta - \theta_0)) \tag{S2}$$

V are the potential energies, k are force constants, r is a bond distance, ϕ is a bond angle, θ is a dihedral angle, and n is the multiplicity of the dihedral.

S1.1 TMAO

S1.1.1 HMKH

The HMKH model[1] parameters for 1 bar are:

Bond type	r_0 (nm)		
O-N	0.1407		
N-C	0.1506		
C-H	0.1082		
Angle type	ϕ_0 (deg)	k_ϕ (kJ mol^{-1} rad^{-2})	
O-N-C	109.99	255.16	
C-N-C	108.16	576.55	
N-C-H	108.07	209.10	
H-C-H	108.25	229.74	
Dihedral type	θ_0 (deg)	k_θ (kJ mol^{-1})	n
O-N-C-H	0	1.13	3
C-N-C-H	0	1.13	3

Table S1: Bond, bond angle, and dihedral parameters of the TMAO HMKH force field.

	O	N	C	H
σ (nm)	0.3266	0.2926	0.3707	0.2130
ϵ (kJ mol^{-1})	0.6389	0.8374	0.2830	0.07745
q (e)	-0.815	0.605	-0.260	0.110

Table S2: Lennard-Jones parameters and partial charges of the TMAO HMKH force field.

S1.1.2 Netz(m)

For the Netz(m) model[2], complete parameters and topology files are available in the SI of ref. [3]. Note that in combination with the KBFF urea model[4], the TMAO-TMAO Lennard-Jones interactions are scaled by 0.9 and the TMAO-urea interactions by 1.1.

S1.2 Urea

S1.2.1 HMKH

The HMKH model[5] notably uses different charges for the hydrogen atoms pointing towards oxygen (H^{cis}) and away (H^{trans}). The parameters are:

Bond type	r_0 (nm)		
C-O	0.12290		
C-N	0.13350		
N-H	0.10100		
Angle type	ϕ_0 (deg)	k_ϕ (kJ mol^{-1} rad^{-2})	
C-N-H	120	390	
H-N-H	120	445	
O-C-N	121.4	730	
N-C-N	117.2	670	
Dihedral type	θ_0 (deg)	k_θ (kJ mol^{-1})	n
O-C-N-H	0.0	8.36800	1
O-C-N-H	180.0	10.46000	2
N-C-N-H	180.0	10.46000	2
N-N-C-O	180.0	43.932	2
C-H-N-H	180.0	4.184	2

Table S3: Bond, bond angle, and dihedral parameters of the urea HMKH force field.

	O	N	C	H^{cis}	H^{trans}
σ (nm)	0.31377	0.34452	0.36039	0.11333	0.11333
ϵ (kJ mol^{-1})	0.59432	0.51114	0.35982	0.065689	0.065689
q (e)	-0.6162	-0.8400	0.6068	0.4026	0.4421

Table S4: Lennard-Jones parameters and partial charges of the urea HMKH force field. H^{cis} (H^{trans}) refer to the hydrogen atoms on the same (opposite) side of the C-N bond as oxygen.

S1.2.2 KBFF

The KBFF model parameters can be found in ref. [4]. Topology files are also available in the SI of ref. [3].

S2 System compositions

System compositions of boxes with $\approx$ 2.5 nm length:

L / nm	N_W	N_T	N_U	c_T/ mol L^{-1}	c_U/ mol L^{-1}	t / μs
2.53823	538	0	0	0	0.0	4
2.54023	485	15	0	1.5	0.0	32
2.55971	438	30	0	3.0	0.0	32
2.55055	478	0	30	0.0	3.0	32
2.54748	415	15	30	1.5	3.0	32
2.55028	357	30	30	3.0	3.0	64
3.21295	714	60	60	3.0	3.0	8

(a)

N_W	N_T	N_U	c_T/ mol L^{-1}	c_U/ mol L^{-1}	t / μs
2000	40	40	1.0	1.0	0.5
463	10	20	1.0	2.0	1.0
1852	40	80	1.0	2.0	0.5
1501	80	160	2.0	4.0	0.5
1540	100	100	2.5	2.5	0.5

(b)

Table S5: System compositions for the calculation of dielectric spectra (a) and activity data (b) with cubic box lengths L, approximate molar concentrations c and total sampling time t.

S3 Finite size effect in dielectric loss spectra

There is no noticeable finite size effect in the components of the dielectric loss.

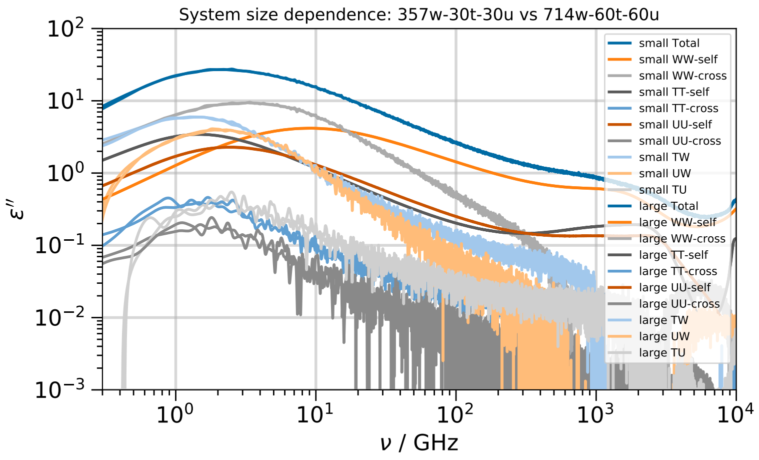

Figure S1: Dielectric loss comparison of 3 M TMAO + 3 M Urea for different system sizes.

S4 Relation between Fourier transforms of dipole and dipole-current autocorrelations

The time autorcorrelation function of the dipole moments $\mathbf{M}$ can be expressed using the convolution theorem

$$\langle \mathbf{M}(0)\mathbf{M}(t)\rangle = \mathcal{F}^{-1}(\mathcal{F}(\mathbf{M}) \cdot \mathcal{F}(\mathbf{M})^*) \tag{S3}$$

since $\mathbf{M}$ is real and therefore $\mathcal{F}(\mathbf{M}(-t)) = \mathcal{F}\left(\mathbf{M}(t)\right)^*$ with the complex conjugate *. Using the following property of Fourier transforms

$$\mathcal{F}(\dot{\mathbf{M}}) = i2\pi\nu\mathcal{F}(\mathbf{M}) \tag{S4}$$

yields

$$\langle \dot{\mathbf{M}}(0)\dot{\mathbf{M}}(t)\rangle = (2\pi\nu)^2\langle \mathbf{M}(0)\mathbf{M}(t)\rangle \tag{S5}$$

S5 Error estimates for KBIs and activity coefficient derivatives in ternary systems

Error estimates for Kirkwood-Buff integrals were determined by dividing the trajectories into 10 blocks, calculating the RDFs and resulting KBIs for each block, and taking the standard deviation of the mean. Under the assumption that KBIs are independent variables, the error estimate of y_{AA} can be propagated from the standard deviations s of the 6 KBIs:

$$s_{y_{AA}} = \sqrt{\sum_{j=1}^{3}\sum_{i=1}^{j}\left(\frac{\partial y_{AA}}{\partial G_{ij}}\right)^2 s_{G_{ij}}^2} \tag{S6}$$

The derivatives of the auxiliary quantities (see main article) with respect to the KBIs G_{ij} are:

$$\frac{\partial \Delta_{AB}}{\partial G_{AA}} \equiv \frac{\partial \Delta_{AB}}{\partial G_{BB}} = 1 \tag{S7}$$

$$\frac{\partial \Delta_{AB}}{\partial G_{AB}} = -2 \tag{S8}$$

$$\frac{\partial \Delta_{AB}}{\partial G_{AC}} \equiv \frac{\partial \Delta_{AB}}{\partial G_{BC}} \equiv \frac{\partial \Delta_{AB}}{\partial G_{CC}} = 0 \tag{S9}$$

$$\frac{\partial \eta}{\partial G_{AA}} = c_A c_B + c_A c_C + c_A c_B c_C \Delta_{BC} \tag{S10}$$

$$\frac{\partial \eta}{\partial G_{AB}} = -2c_A c_B + c_A c_B c_C (\Delta_{AB} - \Delta_{AC} - \Delta_{BC}) \tag{S11}$$

The six expressions for the derivatives of the activity coefficient derivatives y_{AA} with respect to the KBIs are :

$$\frac{\partial y_{AA}}{\partial G_{AA}} = \frac{-(c_B + c_C + c_B c_C \Delta_{BC}) \frac{\partial \eta}{\partial G_{AA}}}{\eta^2} \tag{S12}$$

$$\frac{\partial y_{AA}}{\partial G_{BB}} = \frac{\eta c_B c_C - (c_B + c_C + c_B c_C \Delta_{BC}) \frac{\partial \eta}{\partial G_{BB}}}{\eta^2} \tag{S13}$$

$$\frac{\partial y_{AA}}{\partial G_{CC}} = \frac{\eta c_B c_C - (c_B + c_C + c_B c_C \Delta_{BC}) \frac{\partial \eta}{\partial G_{CC}}}{\eta^2} \tag{S14}$$

$$\frac{\partial y_{AA}}{\partial G_{AB}} = \frac{-(c_B + c_C + c_B c_C \Delta_{BC}) \frac{\partial \eta}{\partial G_{AB}}}{\eta^2} \tag{S15}$$

$$\frac{\partial y_{AA}}{\partial G_{AC}} = \frac{-(c_B + c_C + c_B c_C \Delta_{BC}) \frac{\partial \eta}{\partial G_{AC}}}{\eta^2} \tag{S16}$$

$$\frac{\partial y_{AA}}{\partial G_{BC}} = \frac{-2\eta c_B c_C - (c_B + c_C + c_B c_C \Delta_{BC}) \frac{\partial \eta}{\partial G_{BC}}}{\eta^2} \tag{S17}$$

S5.1 Finite size effect

Notably, the G_{UU} have a systematic finite size effect (fig. S2) that changes the sign. The finite size effect for y_{TT} and y_{UU} for the 1 M TMAO and 2 M Urea systems with a ~2.5 nm and 4 nm box are in table S6. We find that the effects on y_{TT} and y_{UU} are negligible compared to the deviation from the experimental value.

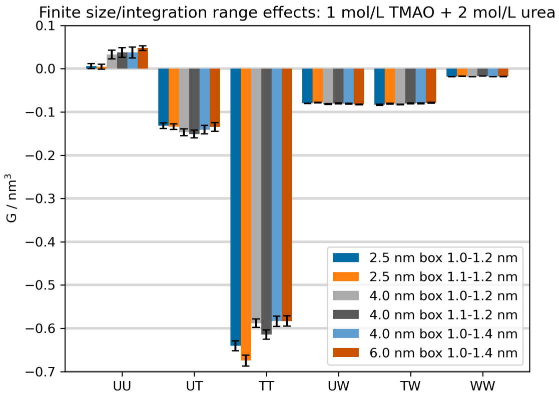

Figure S2: KBI dependence on box size and averaging range (HMKH TMAO//HMKH urea//TIP4P/2005 force fields).

	$L = 2.5$ nm	$L = 4$ nm
y_{TT}	0.387 $\pm$ 0.014	0.324 $\pm$ 0.011
y_{UU}	-0.180 $\pm$ 0.005	-0.204 $\pm$ 0.008

Table S6: Finite size effect for activity coefficient derivatives at 1 M TMAO + 2 M urea for cubic boxes with $L = 2.5$ and 4 nm. Integration range: 1.0-1.2 nm.

References

[1] Christoph Hölzl, Patrick Kibies, Sho Imoto, Roland Frach, Saba Suladze, Roland Winter, Dominik Marx, Dominik Horinek, and Stefan M. Kast. Design principles for high–pressure force fields: Aqueous TMAO solutions from ambient to kilobar pressures. *J. Chem. Phys.*, 144(14):144104, 2016.

[2] Pritam Ganguly, Pablo Boserman, Nico F. A. van der Vegt, and Joan-Emma Shea. Trimethylamine N–oxide Counteracts Urea Denaturation by Inhibiting Protein–Urea Preferential Interaction. *J. Am. Chem. Soc.*, 140(1):483–492, 2018.

[3] Pritam Ganguly, Jakub Polák, Nico F. A. van der Vegt, Jan Heyda, and Joan-Emma Shea. Protein Stability in TMAO and Mixed Urea–TMAO Solutions. *J. Phys. Chem. B*, 124(29):6181–6197, 2020.

[4] Samantha Weerasinghe and Paul E. Smith. A Kirkwood-Buff Derived Force Field for Mixtures of Urea and Water. *J. Phys. Chem. B*, 107(16):3891–3898, 2003.

[5] Christoph Hölzl, Patrick Kibies, Sho Imoto, Jan Noetzel, Michael Knierbein, Paul Salmen, Michael Paulus, Julia Nase, Christoph Held, Gabriele Sadowski, Dominik Marx, Stefan M Kast, and Dominik Horinek. Structure and thermodynamics of aqueous urea solutions from ambient to kilobar pressures : From thermodynamic modeling, experiments, and first principles simulations to an accurate force field description. *Biophys. Chem.*, 254(September):106260, 2019.
